# Author Correction: All-optical spatiotemporal mapping of ROS dynamics across mitochondrial microdomains in situ

**DOI:** 10.1038/s41467-023-44146-6

**Published:** 2023-12-12

**Authors:** Shon A. Koren, Nada Ahmed Selim, Lizbeth De la Rosa, Jacob Horn, M. Arsalan Farooqi, Alicia Y. Wei, Annika Müller-Eigner, Jacen Emerson, Gail V. W. Johnson, Andrew P. Wojtovich

**Affiliations:** 1 University of Rochester Medical Center, Department of Anesthesiology and Perioperative Medicine, 575 Elmwood Ave., Rochester, NY 14642 Box 711/604, USA; 2 University of Rochester Medical Center, Department of Pharmacology and Physiology, 575 Elmwood Ave., Rochester, NY 14642 Box 711/604, USA; 3 Research Group Epigenetics, Metabolism and Longevity, Research Institute for Farm Animal Biology (FBN), Dummerstorf, 18196 Germany

**Keywords:** Optogenetics, Microscopy, Energy metabolism

Correction to: *Nature Communications* 10.1038/s41467-023-41682-z, published online 27 September 2023

In the original version of this Article, there was an error in Figure 3a. The electrons were inadvertently enlarged, obscuring the negative signs also included in the image. This error has now been corrected in both the PDF and HTML versions of the Article.

