# Supplementary material for: All-optical spatiotemporal mapping of ROS dynamics across mitochondrial microdomains in situ
Source: Nat Commun. 2023 Sep 27;14:6036. doi: 10.1038/s41467-023-41682-z (PMC10533892; doi:10.1038/s41467-023-41682-z)
Supplement: Supplementary file 1 — Supplementary Information [file 41467_2023_41682_MOESM1_ESM.pdf]

Supplementary Information

All-optical spatiotemporal mapping of ROS dynamics across mitochondrial microdomains *in situ*

Supplementary Fig. 1 Spatiotemporal manipulation and monitoring of ROS in MEF single mitochondria

Supplementary Fig. 2 KillerRed photostimulation does not significantly alter light-induced mitochondrial depolarization

Supplementary Fig. 3 Photostimulation does not induce HyPer7 oxidation or transient mitochondrial elongation

Supplementary Fig. 4 ROS dynamics and mitochondrial transient elongation is regulated by mitochondrial fission and fusion

Supplementary Fig. 5 Single mitochondrial linescans of microdomain-targeted HyPer7

Supplementary Fig. 6 ROS and pH sensitivity of HyPer7 and ROS-insensitive HyPer7(C121S) variant

Supplementary Fig. 7 Microdomain-specific decay of HyPer7 following exogenous hydrogen peroxide

Supplementary Fig. 8 All-optical mitochondrial ROS generation and detection in live cells with kinetic parameters

Supplementary Fig. 9 Knock-down of endogenous SOD1 and SOD2 alters ROS dynamics

Supplementary Fig. 10 Graphical summary

Supplementary Data 1: Primer list

Supplementary Figure 1

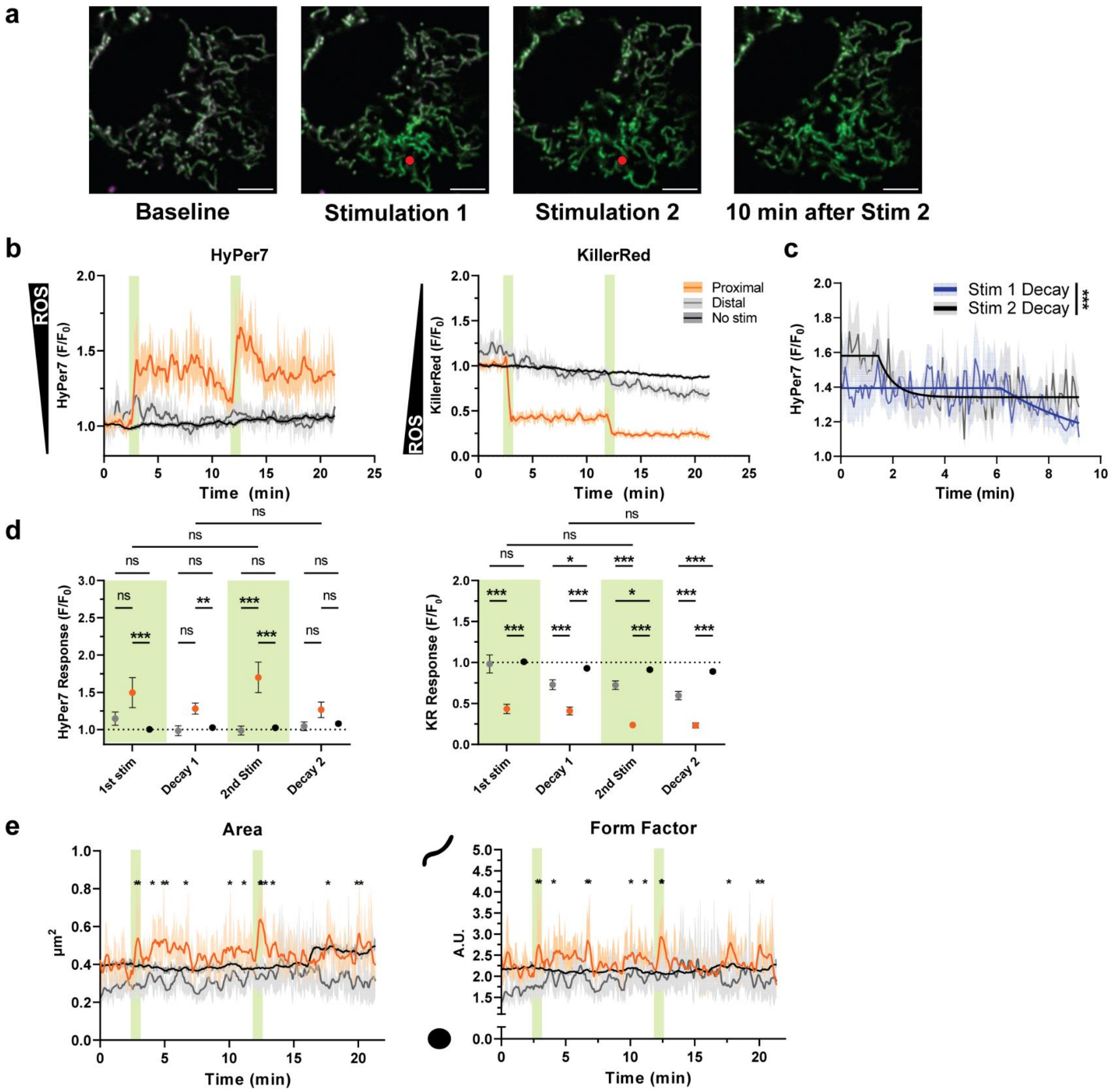

Supplementary Figure 1: Spatiotemporal manipulation and monitoring of ROS in MEF single mitochondria

a Representative image of Matrix-HyPer7 and Matrix-KR photostimulation in MEF cells, merged composite. Scale bar represents 5 µm. Red circle denotes spot photostimulation point. **b** Single mitochondrial responses of HyPer7 and KR intensities normalized to area and baseline, mean ± SEM, N = 20 - 334 mitochondria on average per group. Mitochondria were separated by distance to spot stimulation point. **c** Decay of stimulation 1 and 2 fitted with a nonlinear variable plateau followed by single phase decay, smoothed mean ± SEM, n = 26 - 28 mitochondria on average per condition over at least 3 independent experiments. **d** Comparison of HyPer7 and KR responses at first frame immediately following pulse 1, 10 minutes following first stimulation, immediately following pulse 2, and 10 minutes following second stimulation. Two-way ANOVA with Tukey *post-hoc* multiple comparisons, mean ± SEM. **e** Measured mitochondrial morphological characteristics between subpopulations. Only significant timepoints where proximal measures were significantly different from both distal and no stimulation controls are shown. Two-way ANOVA with Tukey *post-hoc* multiple comparisons, mean ± SEM. Green bars indicate single frames of KR photostimulation. *$p < 0.05$, **$p < 0.01$, ***$p < 0.001$.

Supplementary Figure 2

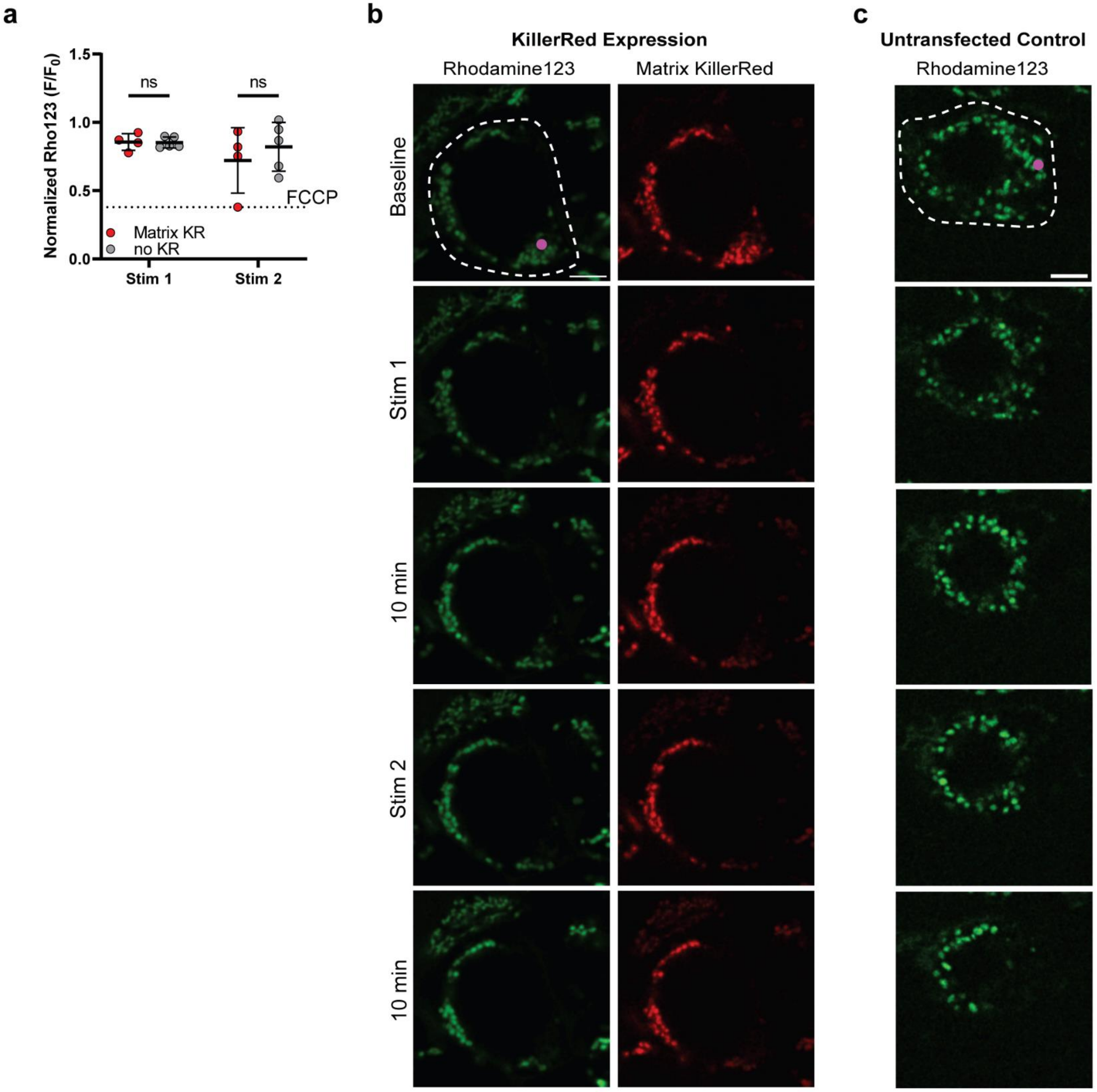

Supplementary Figure 2: KillerRed photostimulation does not significantly alter light-induced mitochondrial depolarization

a HEK293T cells were loaded with Rho123 and photostimulated with 561 nm light twice over 20 min recordings, consistent with spot stimulation KillerRed experiments. Whole-cell Rho123 intensity was normalized to baseline and compared between cells expressing matrix-KR and untransfected cells (4 - 5 cells per group.) Two-way mixed-effects, repeated measure ANOVA with Sidak *post-hoc* multiple comparisons, mean ± SD. Some cells were treated with FCCP to fully depolarize mitochondria to establish the dynamic range of Rho123. **b** Representative images of Matrix-KR (red) expressing cells loaded with Rho123 (green) and photostimulated. Circle denotes spot photostimulation point. **c** As **b**, but without matrix-KillerRed transfection. Scalebar represents 5 µm. Red circle denotes spot photostimulation point.

Supplementary Figure 3

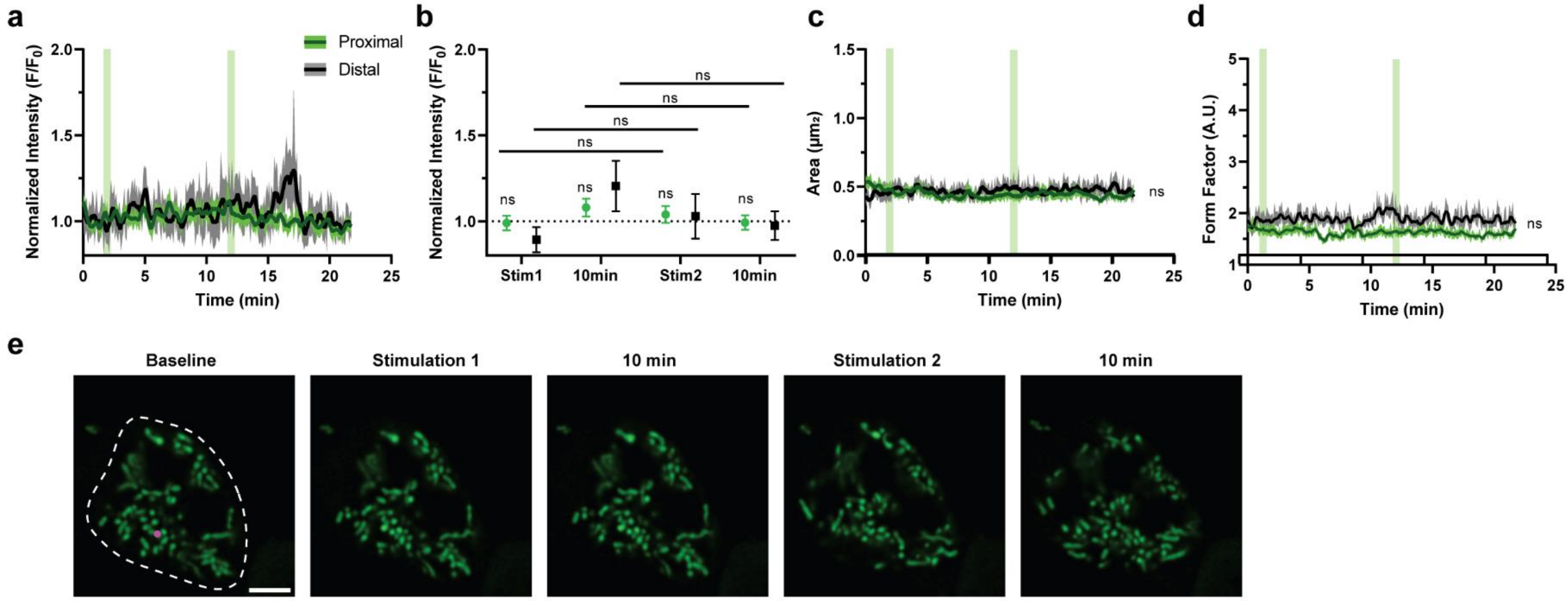

Supplementary Figure 3: Photostimulation alone does not induce HyPer7 oxidation or transient mitochondrial elongation

a Single mitochondrial responses of HyPer7 intensities normalized to area and baseline, separated into proximal and distal distance from the spot stimulation point. **b** Comparison of HyPer7 responses at first frame immediately following pulse 1, 10 minutes following first stimulation, immediately following pulse 2, and 10 minutes following second stimulation. Two-way ANOVA with Tukey *post-hoc* multiple comparisons correction. **c** Measured area between mitochondrial subpopulations over time. Two-way ANOVA with Sidak *post-hoc* multiple comparisons correction. **d** As in **c**, but for mitochondrial form factor. **e** Representative images of matrix-HyPer7 (green) expressing cells throughout the photostimulation protocol. Scalebar represents 5 µm. Red circle denotes spot photostimulation point. Mean ± SEM denoted, N = 55-82 mitochondria on average per group used in each comparison. *$p < 0.05$, **$p < 0.01$, ***$p < 0.001$.

Supplementary Figure 4

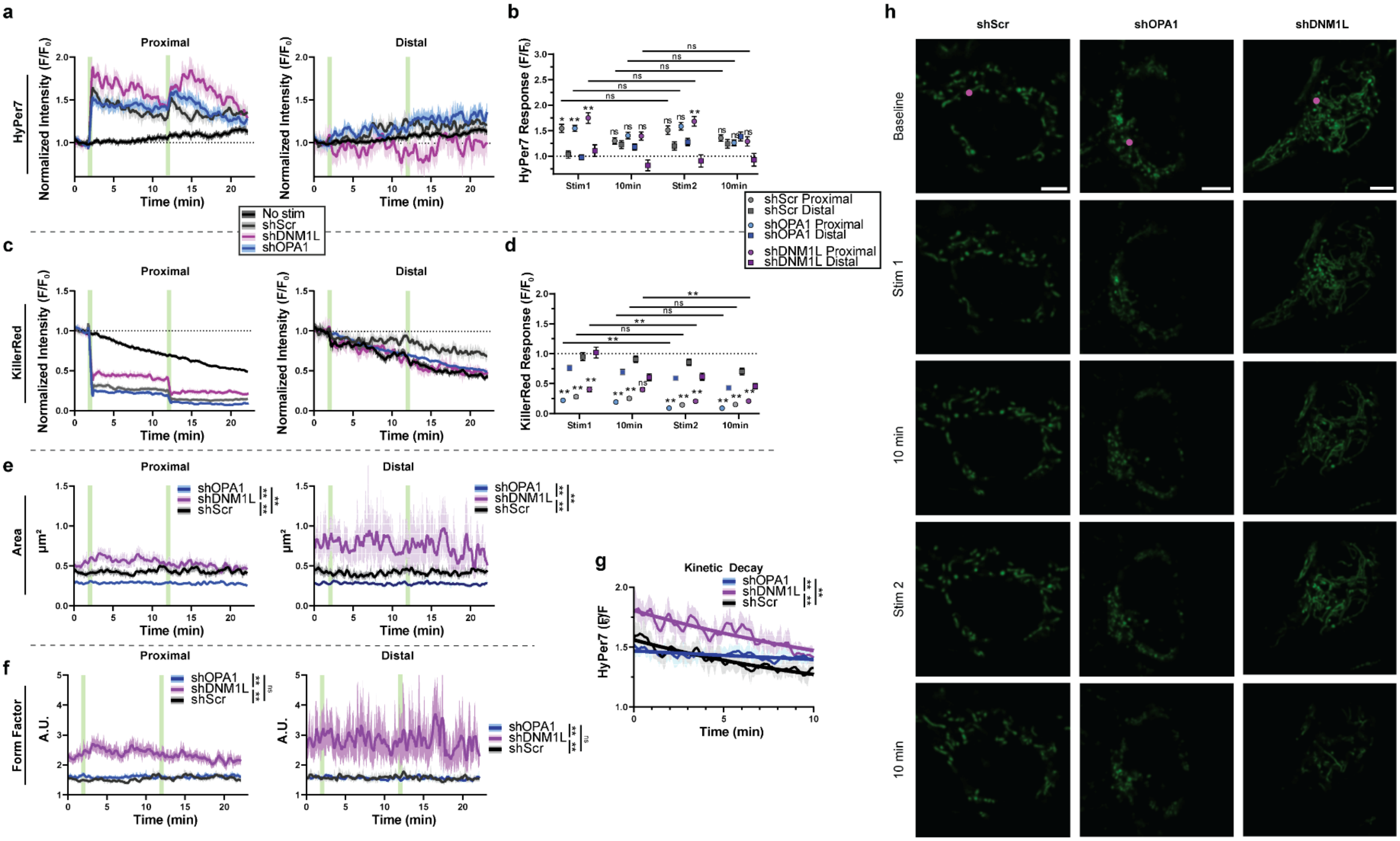

Supplementary Figure 4: ROS dynamics and mitochondrial transient elongation is regulated by mitochondrial fission and fusion

a Single mitochondrial responses of HyPer7 intensities normalized to area and baseline, separated into proximal and distal subpopulations as measured in distance from the spot stimulation point. **b** Comparison of HyPer7 responses at first frame immediately following pulse 1, 10 minutes following first stimulation, immediately following pulse 2, and 10 minutes following second stimulation. Two-way ANOVA with Tukey *post-hoc* multiple comparisons. **c** As in **a**, but for KillerRed intensity. **d** As in **b**, but for KillerRed responses. **e** Measured area between mitochondrial subpopulations over time. Two-way ANOVA with Tukey *post-hoc* multiple comparisons. **f** As **e**, but for form factor. **g** Kinetic analysis of HyPer7 decay rates following KR photostimulation, nonlinear single phase decay, smoothed mean ± SEM. Rate constants compared with one-way ANOVA with Tukey *post-hoc* multiple comparisons, n = 3 - 4 independent experiments. **h** Representative images of each cell line expressing matrix-HyPer7 (green) throughout the photostimulation protocol. Scalebar represents 5 µm. Red circle denotes spot photostimulation point. Mean ± SEM denoted, N = 26 - 152 mitochondria on average per subpopulation in each condition. *p < 0.05, **p < 0.01.

Supplementary Figure 5

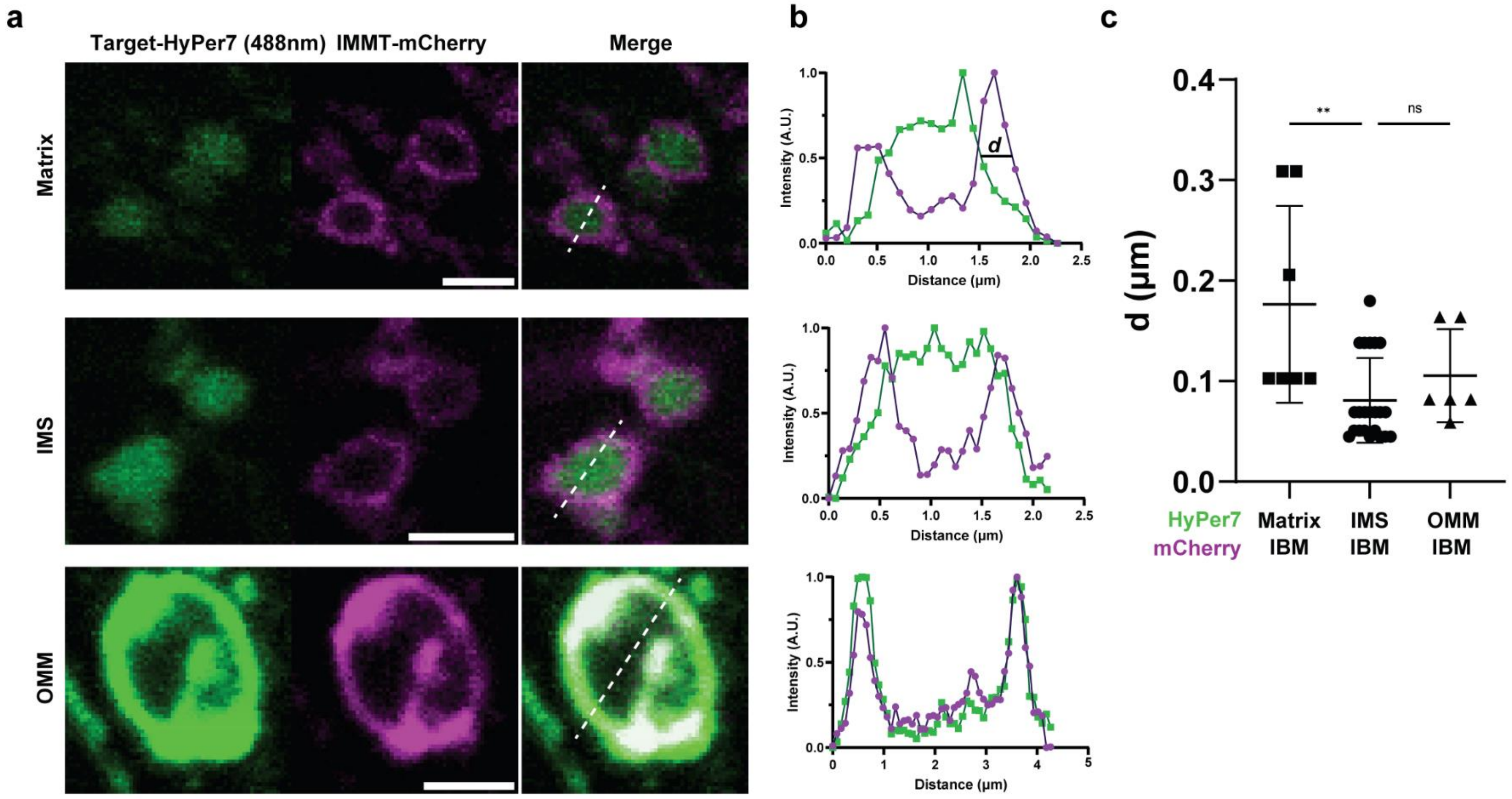

Supplementary Figure 5: Single mitochondrial linescans of microdomain-targeted HyPer7

a Representative image of mitochondrial linescans of matrix, IMS, and OMM-HyPer7 mitochondria (green) in IMMT-mCherry (magenta) expressing *C. elegans.* Scale bar represents 2 µm. **b** Representative linescan data from A indicating distance metric between HyPer7 microdomain and the IBM (IMMT-mCherry). **c** Comparison of distance metric between microdomain-specific HyPer7 and IBM-mCherry, mean ± SD, N = 6 – 22 mitochondria, one-way Kruskal-Wallis ANOVA with Dunn's *post-hoc* correction. $*p < 0.05$, $**p < 0.01$.

Supplementary Figure 6

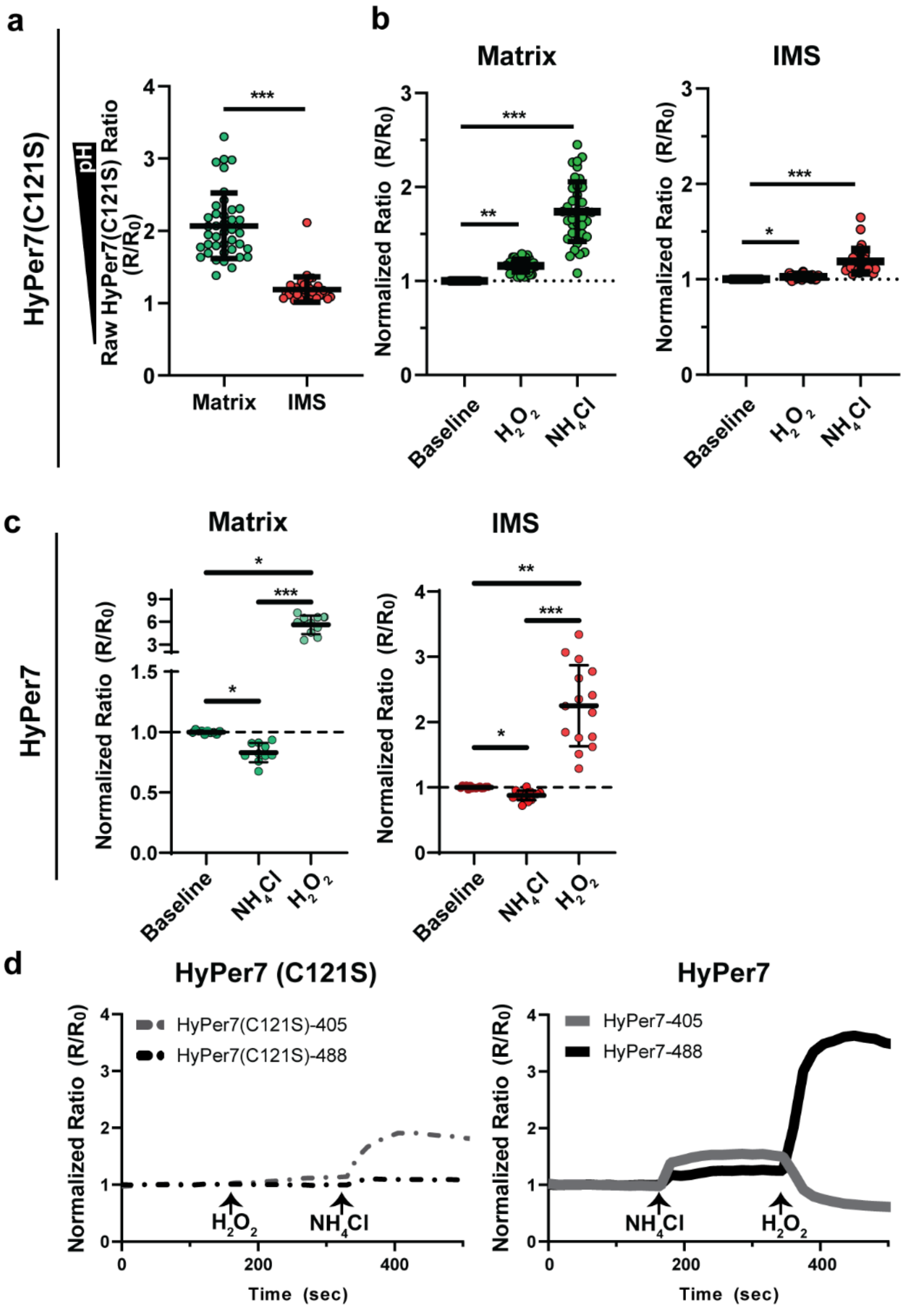

Supplementary Figure 6: ROS and pH sensitivity of HyPer7 and ROS-insensitive HyPer7(C121S) variant

a Raw HyPer7 ratio (488/405 nm excitation) of HyPer7(C121S) mutant targeted to mitochondrial matrix and IMS using two-tailed Mann-Whitney test, mean ± SD, N = 38 - 40 cells. **b** Baseline-normalized HyPer7(C121S) mutant ratio (R/R_0) of matrix- and IMS-targeted HyPer7 in response to saturating 100 µM H_2O_2 and 40 mM NH_4Cl using Kruskal-Wallis one-way ANOVA with Dunn's *post-hoc* correction, mean ± SD, N = 38 - 40 cells. **c** As in **b**, but for HyPer7. N = 10 - 15 cells. **d** Average traces of HyPer7(C121S) mutant and HyPer7 to H_2O_2 and NH_4Cl treatment. *$p < 0.05$, **$p < 0.01$, ***$p < 0.001$.

Supplementary Figure 7

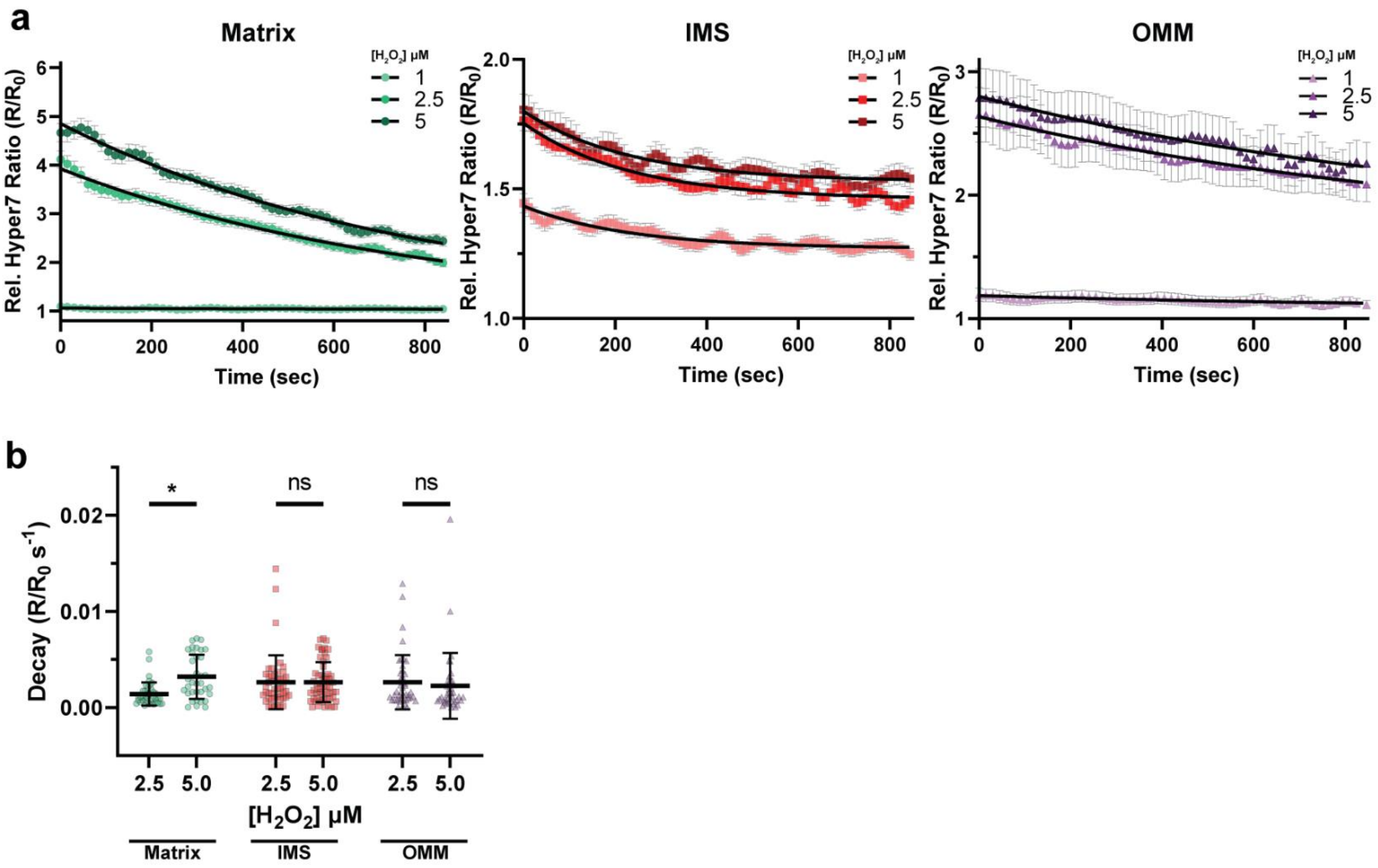

Supplementary Figure 7: Microdomain-specific decay of HyPer7 following exogenous hydrogen peroxide

a Relative HyPer7 ratio decay kinetics fit to nonlinear one-phase decay curves per microdomain after exposure to 1, 2.5, and 5 µM exogenous H_2O_2. N = 35 – 57 cells, mean ± SD. **b** Statistical comparison of decay k constant of microdomain-specific HyPer7 ratio intensity over time after 1, 2.5, and 5 µM H_2O_2 treatment. Two-way ANOVA with Tukey *post-hoc* correction, N = 35 – 57 cells, mean ± SD. *$p < 0.05$

Supplementary Figure 8

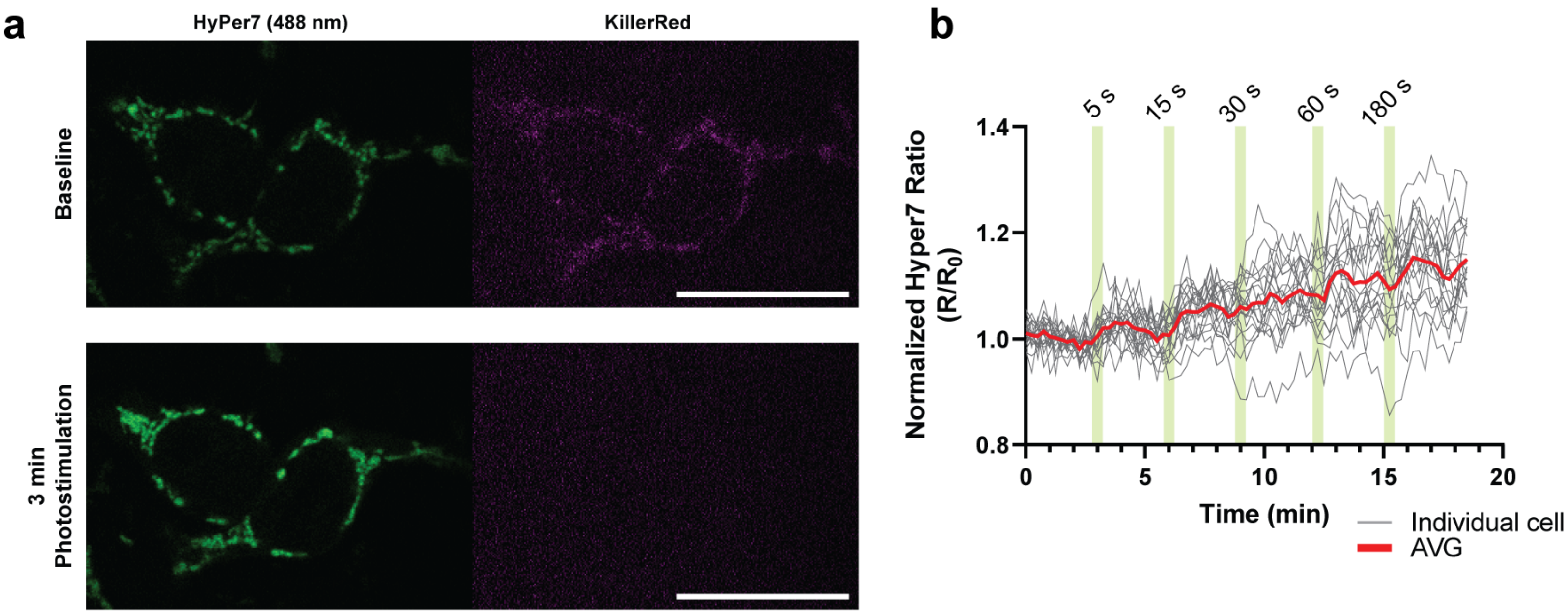

Supplementary Figure 8: All-optical mitochondrial ROS generation and detection in live cells with kinetic parameters

a Representative image of 488 nm excited matrix-HyPer7 (green) and matrix-KR (magenta) in cells before and after 180 sec of whole-cell photostimulation indicating complete photobleaching of KR. **b** Individual cell (grey) and averaged (red) traces of dual excited ratiometric matrix-HyPer7 normalized to baseline (R/R_0) of denoted durations of whole-cell KR photostimulation. Scalebar represents 25 µm.

Supplementary Figure 9

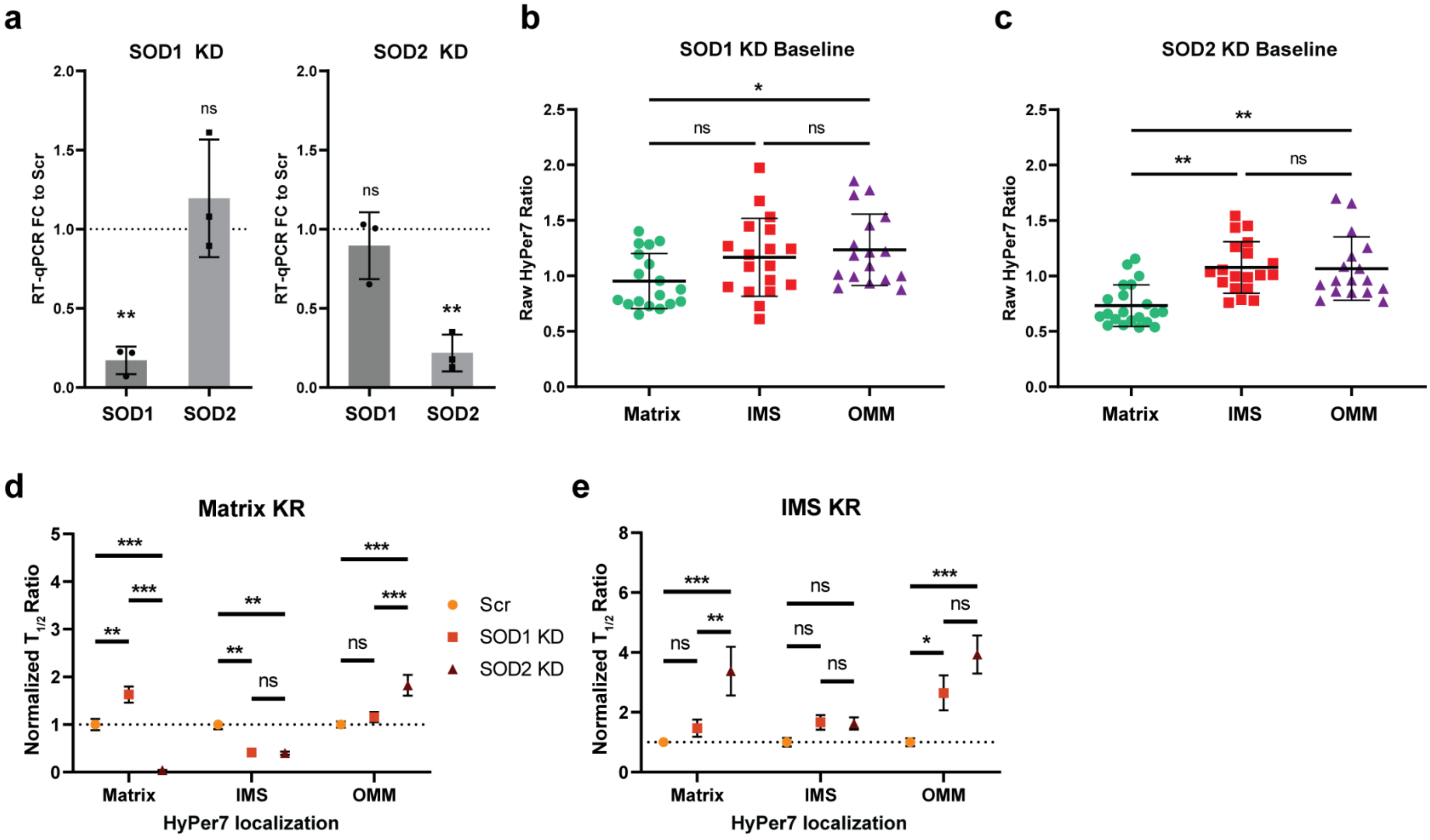

Supplementary Figure 9: Knock-down of endogenous SOD1 and SOD2 alters ROS dynamics

a RT-qPCR fold change levels of SOD1 and SOD2 in either SOD1 or SOD2 KD cell lines, normalized to *HPRT* and compared to Scr control cells. N = 3 independent experiments, mean ± SD. **b** Absolute HyPer7 ratio per microdomain at baseline in SOD1 KD cells. N = 17 – 18 cells across at least two fields of view over two independent experiments. One-way ANOVA with Tukey *post-hoc* multiple comparisons. **c** As in **b**, but for SOD2 KD cells. N = 17 – 21 cells across at least two fields of view over two independent experiments, mean ± SD. One-way ANOVA with Tukey *post-hoc* multiple comparisons. **d** Time to half maximum ($T_{1/2}$) for microdomain-targeted HyPer7 and matrix-KR experiments. Mean ± SEM, N = 12 – 21 cells across at least two independent experiments. Two-way ANOVA with Tukey *post-hoc* correction. **e** As in **d**, but for IMS-KR experiments. Mean ± SEM, N = 7 – 14 cells across at least two independent experiments. Two-way ANOVA with Tukey *post-hoc* correction.

Supplementary Figure 10: Graphical summary

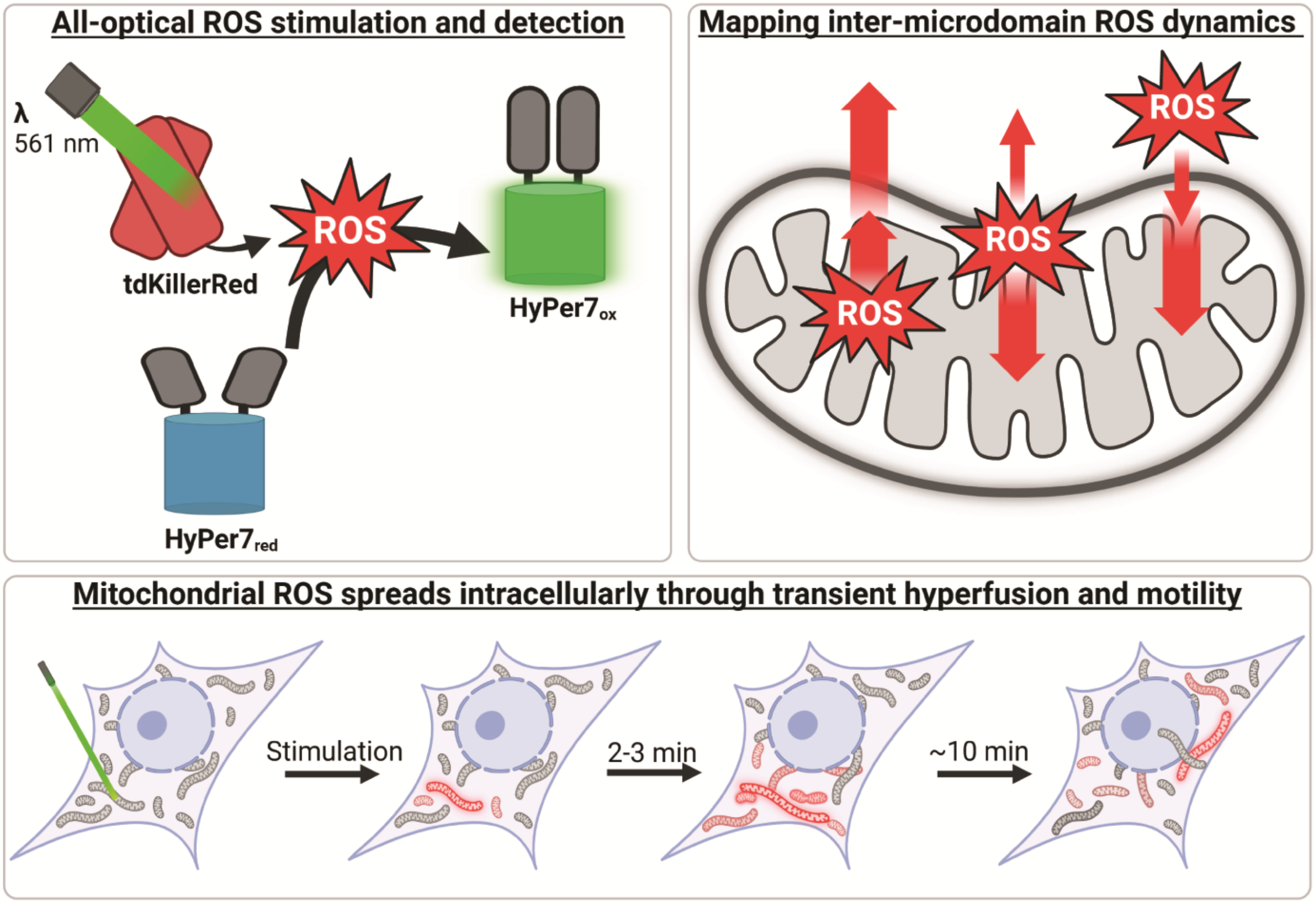

Supplementary Figure 10: Graphical summary

Schematic summarizing all-optical techniques used in this study to generate and measure ROS across mitochondrial microdomains, as well as ROS-mediated transient mitochondrial hyperfusion and motility. Created with BioRender.com.

Supplementary Data 1: Primer list

**IVA**						
**Model**	**Protein Expressed**	**Final Construct**	**Insert Template Plasmid**	**Insert Primer Set**	**Vector Template Plasmid**	**Vector Primer Set**
Cells	Matrix-KR	pJH1 (CMV::Cox8Ax2::tdKR)	pArrestRed	FWD: ccattcgttgggggataccggtGCCACCCTCGAGGCCACCATG	pCS2::CMV::Cox8Ax2::Hyper7	FWD: ctcgagcctctagaactatag
				REV: actatagttctagaggctcgagTCTAGAGTCGCGGCCGCTTA		REV: accggtatcccccaacgaatg
Cells	IMS-KR	pJH2 (CMV::Smac::tdKR)	pArrestRed	FWD: actatagttctagaggctcgagTCTAGAGTCGCGGCCGCTTA	pCS2::CMV::Smac::Hyper7	FWD: ctcgagcctctagaactatag
				REV: ggatccacccgtcgccaccggtGCCACCCTCGAGGCCACCATG		REV: accggtggcgacgggtggatc
Cells	OMS-KR	pJH3 (CMV::TOM20::tdKR)	pAJT1 (eft3::TOM20N::tdKillerRed)	FWD: tcccatcgattcgaattcgccaccATGTCGGACACAATTCTTGG	pJH2 (pCS2::CMV::Smac::tdKR)	FWD: GCCACCCTCGAGGCCACCATG
				REV: CCATGGTGGCCTCGAGGGTGGCTGCTCCAGCCTGGGCACGTC		REV: ggtggcgaattcgaatcgatg
Cells	IBM-HyPer7	pJH4 (CMV::IMMT::HyPer7)	pAJT3 (eft3::IMMT::tdKillerRed)	FWD: ccatcgattcgaattcgccaccATGCTGCGGGCCTGTCAGTT	pCS2::CMV::Cox8Ax2::Hyper7	FWD: atccattcgttgggggataccg
				REV: cggtatcccccaacgaatggatTTCCTCTGTGGTTTCAGACG		REV: ggtggcgaattcgaatcgatgg
Cells	OMS-HyPer7	pJH5 (CMV::TOM20::HyPer7)	pAF2 (eft3::TOM20N::HyPer7)	FWD: ccatcgattcgaattcgccaccATGTCGGACACAATTCTTGG	pCS2::CMV::Cox8Ax2::Hyper7	FWD: atccattcgttgggggataccg
				REV: cggtatcccccaacgaatggatTGCTCCAGCCTGGGCACGTC		REV: ggtggcgaattcgaatcgatgg
Worms	Matrix-HyPer7	pNG1 (eft3::2xCox8A::Hyper7::unc54)	pCS2::CMV::Cox8Ax2::Hyper7	FWD: TCCACCGGTCGCCCCTGCAGGccacctggctaatgaggagcaaaacgaattgg	pAJT1 (eft3::TOM20N::tdKR ::unc54)	FWD: GCGGCCGCAATTCCAACTGAGC
				REV: GCTCAGTTGGAATTGCGGCCGCtcaatcgcagatgaagctaacaccatgcag		REV: gCCTGCAGGGGCGACCGGTGGA
Worms	IMS-HyPer7	pAF1 (eft3::Smac::HyPer7::unc54)	pCS2::CMV::IMS::Hyper7	FWD: AACACTTTGCTCtctagaAAAAatggcggctctgaagagttg	pBB20 (eft3::IMMT::MAC::unc54)	FWD: GCGGCCGCAATTCCAACTG
				REV: GCTCAGTTGGAATTGCGGCCGCTCAATCGCAGATGAAGCTAA		REV: TTTTTCTAGAGAGCAAAGTG
Worms	OMS-Hyper7	pAF2 (eft3::TOM20N::HyPer7::unc54)	pCS2::CMV::Cox8Ax2::Hyper7	FWD: CCAGGCTGGAGCACCTGCAGGcCACCTGGCTAATGAGGAGCAAAACGAATTGg	pAJT1 (eft3::TOM20N::tdKR ::unc54)	FWD: GCGGCCGCAATTCCAACTGAG
				REV: GCTCAGTTGGAATTGCGGCCGCTCAATCGCAGATGAAGCTAACACCATGCAG		REV: GCCTGCAGGTGCTCCAGCCTG

Point Mutagenesis:

Matrix-HyPer7(C121S)

Fwd: ctgaccgagggcaactctatgcgtgaccaggtg
Rev: cacctggtcacgcatagagttgccctcggtcag

IMS-HyPer7(C121S)

Fwd: ctgaccgagggcaactctatgcgtgaccaggtg

Rev: cacctggtcacgcatagagttgccctcggtcag

qPCR-RT Primers:

SOD1

Fwd: ACTGGTGGTCCATGAAAAAGC

Rev: AACGACTTCCAGCGTTTCCT

SOD2

Fwd: CTGGACAAACCTCAGCCCTAAC

Rev: AACCTGAGCCTTGGACACCAAC

HPRT

Fwd: CCTGGCGTCTGATTAGTGAT

Rev: AGACGTTCAGTCCTGTCCATAA
