# Supplementary material for: All-optical spatiotemporal mapping of ROS dynamics across mitochondrial microdomains in situ
Source: Nat Commun. 2023 Sep 27;14:6036. doi: 10.1038/s41467-023-41682-z (PMC10533892; doi:10.1038/s41467-023-41682-z)
Supplement: Supplementary file 3 — Description of Additional Supplementary Files [file 41467_2023_41682_MOESM3_ESM.pdf]

Description of Additional Supplementary Information

All-optical spatiotemporal mapping of ROS dynamics across mitochondrial microdomains *in situ*

File name: Supplementary Movie 1
Description: Matrix HyPer7 and KillerRed Photostimulation

File name: Supplementary Movie 2
Description: No stimulation control Matrix HyPer7 and KillerRed

File name: Supplementary Movie 3
Description: Matrix ROS induces motility of oxidized mitochondria

File name: Supplementary Movie 4
Description: Matrix ROS induces transient hyperfusion of oxidized mitochondria
