# Supplementary material for: All-optical spatiotemporal mapping of ROS dynamics across mitochondrial microdomains in situ
Source: Nat Commun. 2023 Sep 27;14:6036. doi: 10.1038/s41467-023-41682-z (PMC10533892; doi:10.1038/s41467-023-41682-z)
Supplement: Supplementary file 8 — Reporting Summary [file 41467_2023_41682_MOESM8_ESM.pdf]

nature portfolio

Corresponding author(s): Wojtovich

Last updated by author(s): 07-11-2023

Reporting Summary

Nature Portfolio wishes to improve the reproducibility of the work that we publish. This form provides structure for consistency and transparency in reporting. For further information on Nature Portfolio policies, see our Editorial Policies and the Editorial Policy Checklist.

Statistics

For all statistical analyses, confirm that the following items are present in the figure legend, table legend, main text, or Methods section.

n/a	Confirmed	
☐	☒	The exact sample size (*n*) for each experimental group/condition, given as a discrete number and unit of measurement
☐	☒	A statement on whether measurements were taken from distinct samples or whether the same sample was measured repeatedly
☐	☒	The statistical test(s) used AND whether they are one- or two-sided<br>*Only common tests should be described solely by name; describe more complex techniques in the Methods section.*
☐	☒	A description of all covariates tested
☐	☒	A description of any assumptions or corrections, such as tests of normality and adjustment for multiple comparisons
☐	☒	A full description of the statistical parameters including central tendency (e.g. means) or other basic estimates (e.g. regression coefficient) AND variation (e.g. standard deviation) or associated estimates of uncertainty (e.g. confidence intervals)
☐	☒	For null hypothesis testing, the test statistic (e.g. *F*, *t*, *r*) with confidence intervals, effect sizes, degrees of freedom and *P* value noted<br>*Give P values as exact values whenever suitable.*
☒	☐	For Bayesian analysis, information on the choice of priors and Markov chain Monte Carlo settings
☒	☐	For hierarchical and complex designs, identification of the appropriate level for tests and full reporting of outcomes
☒	☐	Estimates of effect sizes (e.g. Cohen's *d*, Pearson's *r*), indicating how they were calculated

Our web collection on statistics for biologists contains articles on many of the points above.

Software and code

Policy information about availability of computer code

Data collection	Images were taken using Nikon NIS-Elements AR version 5.42.01 build 1793.
Data analysis	Microsoft excel 2019, R (v4.0.3), ImageJ/FIJI (v1.5.3) with Weka segmentation (v 3.3) and TrackMate(v7) plugins, and Graphpad Prism 9.

For manuscripts utilizing custom algorithms or software that are central to the research but not yet described in published literature, software must be made available to editors and reviewers. We strongly encourage code deposition in a community repository (e.g. GitHub). See the Nature Portfolio guidelines for submitting code & software for further information.

Data

Policy information about availability of data

All manuscripts must include a data availability statement. This statement should provide the following information, where applicable:

- Accession codes, unique identifiers, or web links for publicly available datasets
- A description of any restrictions on data availability
- For clinical datasets or third party data, please ensure that the statement adheres to our policy

All the data supporting the findings are available in the paper and the supplementary information. C. elegans strains and data are available upon request. Plasmids generated by this manuscript are available upon request. Upon a sufficient number of requests, plasmids will be deposited on Addgene.

Human research participants

Policy information about studies involving human research participants and Sex and Gender in Research.

Reporting on sex and gender	N/A, manuscript did not use human research participants.
Population characteristics	N/A, manuscript did not use human research participants.
Recruitment	N/A, manuscript did not use human research participants.
Ethics oversight	N/A, manuscript did not use human research participants.

Note that full information on the approval of the study protocol must also be provided in the manuscript.

Field-specific reporting

Please select the one below that is the best fit for your research. If you are not sure, read the appropriate sections before making your selection.

☒ Life sciences ☐ Behavioural & social sciences ☐ Ecological, evolutionary & environmental sciences

For a reference copy of the document with all sections, see nature.com/documents/nr-reporting-summary-flat.pdf

Life sciences study design

All studies must disclose on these points even when the disclosure is negative.

Sample size	For some experiments, sample size was calculated using a power analysis (StatMate, GraphPad). For others, sample sizes were determined using previous publications (Berry 2020a, Berry 2020b, Trewim 2019).
Data exclusions	No data were excluded from the study, except in TMRM response curve measurements where a 1% ROUT test was done and excluded 9 out of 420 cells. Outlier exclusion had no effect on statistical comparisons.
Replication	Each experiment is composed of multiple replicates as denoted in each figure.
Randomization	Brightly fluorescent cells after biosensor transfection and worms without gross mitochondrial morphology deformations were imaged as described in the Methods section. Random fields of view were otherwise used for long-term imaging and optical experimentation.
Blinding	Experimenters were not blinded to conditions as it would not affect recording live imaging data.

Reporting for specific materials, systems and methods

We require information from authors about some types of materials, experimental systems and methods used in many studies. Here, indicate whether each material, system or method listed is relevant to your study. If you are not sure if a list item applies to your research, read the appropriate section before selecting a response.

Materials & experimental systems

n/a	Involved in the study	
☒	☐	Antibodies
☐	☒	Eukaryotic cell lines
☒	☐	Palaeontology and archaeology
☐	☒	Animals and other organisms
☒	☐	Clinical data
☒	☐	Dual use research of concern

Methods

n/a	Involved in the study	
☒	☐	ChIP-seq
☒	☐	Flow cytometry
☒	☐	MRI-based neuroimaging

Eukaryotic cell lines

Policy information about cell lines and Sex and Gender in Research

Cell line source(s)	HEK293T cells were purchased from System Biosciences and MEFs were purchased from ATCC.

Authentication	Cell lines were authenticated by the suppliers and by visual morphology prior to experiments.
Mycoplasma contamination	Cells tested negative for mycoplasma contamination.
Commonly misidentified lines (See ICLAC register)	No commonly misidentified lines were used in this study.

Animals and other research organisms

Policy information about studies involving animals; ARRIVE guidelines recommended for reporting animal research, and Sex and Gender in Research

Laboratory animals	Caenorhabditis elegans mid-L4 stage were used in this study.
Wild animals	The study did not involve wild animals.
Reporting on sex	Only hermaphrodite worms were used in this study.
Field-collected samples	The study did not involve field-collected samples
Ethics oversight	C. elegans is an invertebrate. Experiments were approved by the University of Rochester Institutional Biosafety Committee.

Note that full information on the approval of the study protocol must also be provided in the manuscript.
